# Comparison of blended learning and traditional lecture method on learning outcomes in the evidence-based medicine course: a comparative study

**DOI:** 10.1186/s12909-024-05659-w

**Published:** 2024-06-20

**Authors:** Kui Liu, Shuang Liu, Yifei Ma, Jun Jiang, Zhenhua Liu, Yi Wan

**Affiliations:** 1 Department of Health Service, Air Force Medical University, Xi’an, Shaanxi 710032 China; 2 Office of Academic Affairs, Air Force Medical University, Xi’an, Shaanxi 710032 China

**Keywords:** Blended-learning, Evidence-based medicine, Comparative study

## Abstract

**Background:**

Blended learning comprised with flipped classroom (FC) and “internet plus” is a new learning strategy that reverses the position of teacher and students in class, and provides abundant learning resources before and after class. This study aimed to assess the impact of blended learning on learning outcomes in evidence-based medicine course, and compare with traditional learning method.

**Methods:**

The participants of the two groups were from two difference cohorts in Air force medical university in China. The two groups toke the same pre-test before class and then were given the teaching of same chapters of evidence-based medicine with two different learning strategy. In the blended learning group, the participants were required to create a debriefing slide about their learning outcomes and the answers of questions given in advance after study the learning material sent by teacher a week before class, and the teacher gave a detailed summary based on the common problems, and distributed multimedia resources for review. After the experiment was carried out, learning outcomes including mastering knowledge, learning satisfaction, and self-evaluation were compared.

**Results:**

37 and 39 participants were enrolled to blended learning and traditional learning groups, respectively, and no statistically significant difference were found in baseline information and pre-test grades. Statistically significant differences were found in learning outcomes including post-test score (*t* = 2.90, *p* = 0.005), changes of scores between pre-test and post-test (*t* = 2.49, *p* = 0.022), learning satisfaction (*t* = 12.41, *p* = 0.001), and self-evaluation of the two groups (*t* = 7.82, *p* = 0.001). Especially, the changes of scores between pre-test and post-test of blended learning and traditional learning groups were 4.05 (4.26), and 2.00 (2.85), respectively.

**Conclusions:**

This study showed that compared with traditional learning strategy, blended learning can effectively enhanced participants’ acquisition of knowledge, learning satisfaction, and self-evaluation in evidence-based medicine. Using blended learning method including “internet plus” and flipped classroom is recommended in the teaching of evidence-based medicine course.

## Background

Evidence-based medicine has played a prominent role in public health and basic medical research, including exploring the risk factors of diseases [1], early diagnosis of disease [2], proper and rational treatment of disease [3], and judgment of disease prognosis [4]. Therefore, evidence-based medicine is an indispensable course, which covers 5 main steps for applying it to clinical practice: defining a clinically relevant question, searching for the best evidence, critically appraising the evidence, applying the evidence, and evaluating the performance of evidence-based medicine [5]. Evidence-based medicine demands practitioner’s solid theoretical knowledge and skilled application ability for it, which puts forward high requirements for the course teaching. Through the study of this course, the graduate students should not only master the basic theory of evidence-based medicine, but also master the thought and method of evidence-based medicine to lay a foundation for their future application in clinical practice. Unfortunately, current medical postgraduate education mainly focuses on learning clinical expertise, which leaves inadequate time to learn evidence-based medicine. Consequently, it is important to develop an effective learning strategy for the evidence-based medicine course to allow medical postgraduates to master knowledge in limited classroom time.

However, the traditional instructional approach also known as lecture-based learning is a passive format learning [6], which mainly relies on lectures from teachers to transfer knowledge, and students only accept the knowledge passively [7]. For medical courses, which involves the acquisition of a large quantity of knowledge [8], the learning result achieved by lecture-based method is far from the expected goal and the requirement of professional work. For medical postgraduates, practical abilities of evidence-based medicine are of great importance, while lecture-based learning did not provide them with any opportunity for practical application of theoretical knowledge but only homework on paper to do [7]. Moreover, critical thinking ability, problem-solving ability and integrated thinking abilities are essential for evidence-based medicine, and it has been proved that lecture-based learning strategies are insufficient in training these abilities [9–11]. In summary, previous studies have proved that knowledge transfer is poor during passive format learning, result in no need to keep traditional teaching and learning strategies in medical education. Therefore, to overcome the disadvantages of the lecture-based learning method, the implementation of new learning strategies with active participation of learners and more innovative methods are required in the teaching process of evidence-based medicine of postgraduates.

Flipped classroom (FC) approach reverses the position of teacher and student in class, in which students acquire basic knowledge though self-learning before class, and apply the knowledge to solve problems proposed by teachers with individual homework or group activities, then report on the result of learning and problem solving and apply the acquired knowledge to solve practical problems under the guidance of the instructor in class [12–15]. Recently, there are more and more implantation of the FC approach in health care course education [16]. Students who attend flipped classroom gave highly positive response on motivation of learning, engagement in learning, and learning satisfaction [17]. However, there are also studies found that FC did not improve learning competence, such as Ilic et al. [18] applied FC approach in evidence-based medicine course, and the experimental group did not achieve higher score as expected. Therefore, further investigations are demanded to evaluate the impact of FC approach in evidence-based medicine course.

The “internet plus” is not an independent learning or teaching method, instead, it’s a combination of internet technology and the process of teaching and learning. For example, during the COVID-19 pandemic, lots of medical universities started online classes by actively preparing for teaching online, owing to the lockdowns, travel restrictions, and quarantines to control the spread of the pandemic [19–21]. Yu-Xin Cao et al. explored lemology teaching with “internet plus” flipped classroom pedagogy with clinical medicine students, and proved that the pedagogy boosted students’ theory learning ability, case analysis ability, and learning satisfaction [22]. Therefore, this comparative study aimed to assess the impact of blended learning comprised with “internet plus” and flipped classroom on learning outcomes in evidence-based medicine course, and compare with lecture-based method to provide reference for full implementation of the new teaching method.

## Methods

### Study participants

To comprehensively evaluate the effects of “internet plus” FC on medical postgraduates, the participants of the study were the postgraduates of a medical university from the 2022–2023 cohort, who majored in multiple disciplines pertain to medical specialty. And the students were assigned into two groups: students form 2022 cohort were allocated into control group which conducted with lecture-based learning, and students from 2023 cohort were allocated into experimental group which conducted with blended learning, “internet plus” flipped classroom. It is worth mentioning that although the two groups were not contemporaneous controls, they were both from the first year of graduate students, it is therefore reasonable to assume that the two groups of students have the same level of knowledge. The inclusion criteria were as follows: voluntary participations who were informed of the objective of the study in advance, have completed professional basic courses of their own majors, and finished the first chapter of the class: Introduction of evidence-based medicine. Finally, 76 students were included in this study, 37 students for blended learning group, and 39 students for lecture-based learning group. The sample size was calculated 31 students for each group using G power 3.1.9 with significance level α = 0.05, effect size (ρ) = 0.70, and power = 0.85. The study was approved by the Ethics Committee of Air Force Medical University (KY20222232-C-1).

### Study design

This study chose the same sections from evidence-based medicine course as learning content, including “How to identify and raise a question in clinical practice”, “Classification, quality, grading and recommendation of evidence”, and “The source and retrieval of the evidence”, which covered the main content of the basic knowledge of evidence-based medicine. Therefore, a high degree of consistency was also maintained in terms of the content of the lectures and the teaching staff, which also made sure the balance between the two groups. Both groups took pre-test before class, and post-test, learning satisfaction, and self-evaluation questionnaire two weeks after the class.

The control group was taught with traditional learning method, as known as lecture-based method. The teacher first gave a lecture about theoretical knowledge according to the specific requirements of syllabus, students answered the questions raised by teacher in class, and took notes. Before the end of the class, the teacher gave a brief summary of the content of the chapter. After class, students completed homework as required, and submitted it three days after class.

**Fig. 1:**
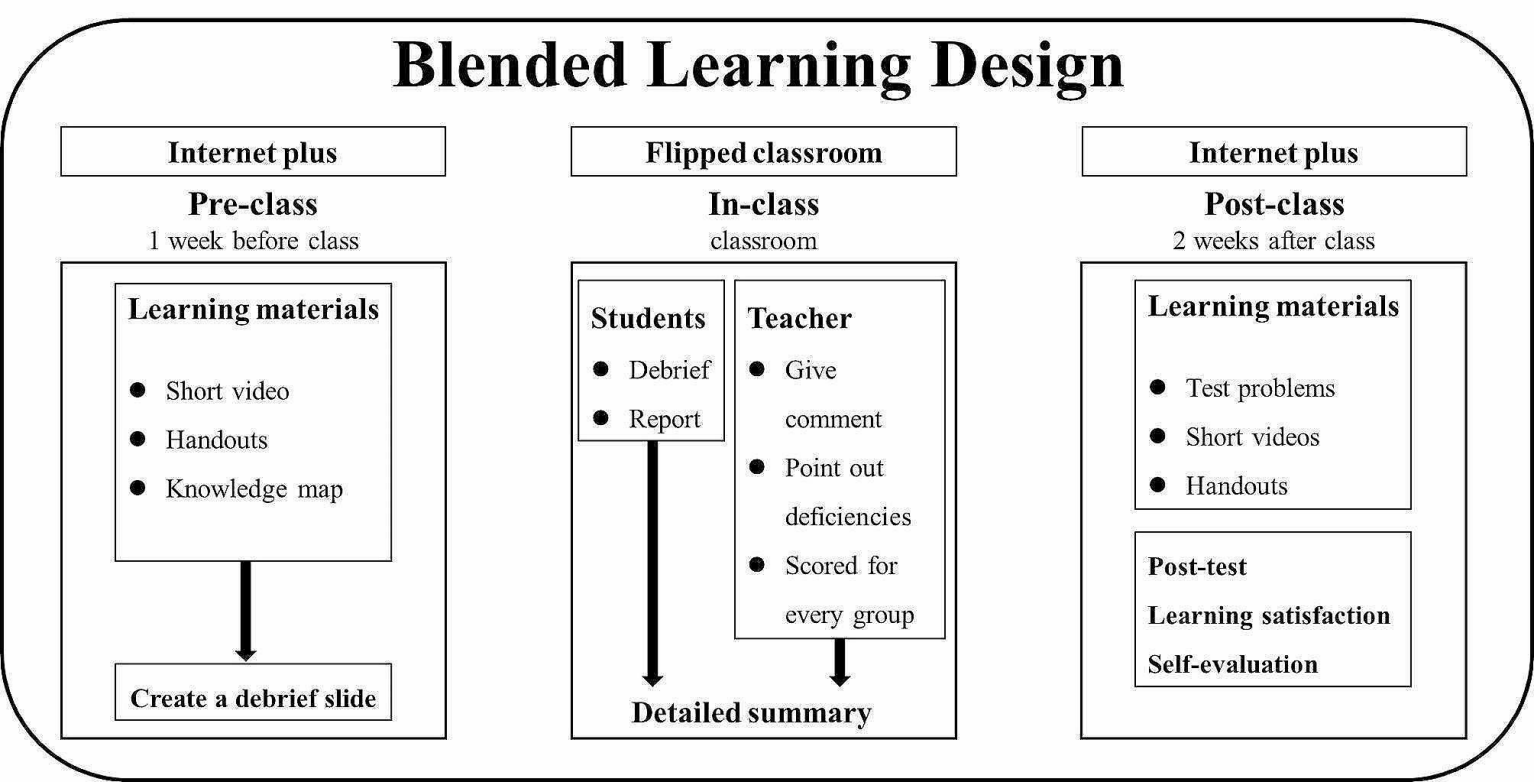
The blended learning design including “internet plus” and FC used in this study

The experimental group used the blended learning comprised of “internet plus” and FC. One week before the classroom session, a learning material including short videos, handouts, and knowledge maps about the lecture was sent to students with several questions about the content. And students were required to formed into 5 subgroups by themselves, and each subgroup needed to create a debriefing slide about their learning outcomes and the answers of questions given in advance. After each group finished their debrief, the instructor gave a brief comment on their report, point out deficiencies in the report, and scored for it according to content integrity, response to questions, production of slides, and fluency of presentation. And before the end of the class, the instructor gave a detailed summary, that mainly focused on the problems existing commonly in the reports, and provided multimedia resources for review, consolidation, and extension. Then the students submitted their homework three days after the class. Figure 1 depicts the blended learning design of this study.

### Effectiveness assessment

After the teaching and learning process of the two groups was completed, a comprehensive assessment including mastering knowledge, learning satisfaction, and self-evaluation were implemented.

The situation of mastering theoretical knowledge was evaluated by post-test after two weeks of the class, which examined the same knowledge as pre-test. The examination with 10 indefinite choice questions were assigned by the instructor according to the syllabus.

A questionnaire with fourteen questions that focused on learning satisfaction was developed by the Graduate School of Air Force Medical University. This scale comprises 14 items to be answered, each of them has 5 points ranging from 1 = strongly disagree to 5 = strongly agree (Table 1). The Cronbach alpha coefficient and the KMO coefficient of the questionnaire were 0.759 and 0.696, respectively, and the Bartlett’s test of sphericity indicated that the questionnaire were with enough construct validity (*P* < 0.001). After students completed the scale, the average score was calculated, and higher score represents better learning satisfaction.

**Table 1:** Questionnaire of students’ learning satisfaction

Categories	
1	Do you consider the class content is easy to understand?
2	Do you consider the class content is helpful for your major learning?
3	How do you think the content of the class is interesting?
4	Do you consider yourself actively participate in class activities?
5	Is the class teaching process carried out according to the plan?
6	Are you satisfied with the teaching methods used in this class?
7	Are you satisfied with the materials used in this class?
8	Do you consider the evaluation method of this class is appropriate?
9	Do you consider the teaching environment (in class) of this class is appropriate?
10	Do you consider the teaching environment (outside class) of this class is appropriate?
11	Do you consider the self-directed learning method adopted in this class is appropriate?
12	Are you satisfied with the detailed design of this class?
13	Are you satisfied with the teacher-student interaction in this class?
14	Do you consider your learning initiative has improved through this class?

Furthermore, to measure the difference of the improvement of the capacity for scientific research between two groups, we developed a questionnaire that evaluated student’s ability by their own. The questionnaire were with 5 items that mainly focused on the ability of find, analyze, and solve scientific problems, including the following questions: (1) Do you think your ability to find scientific problems has improved through the study of this course; (2) Do you think your ability to analyze scientific problems has improved through the study of this course; (3) Do you think your ability to solve scientific problems has improved through the study of this course; (4) Do you think your ability to engage in critical thinking has improved through the study of this course; (5) Do you think your ability of independent learning has improved through the study of this course. Each question has 5 points ranging from 1 = strongly disagree to 5 = strongly agree. Using this scale, the average score was calculated, and higher score represents better self-improvement.

### Statistical analysis

Demographic baseline data and scores of examinations and questionnaire from two groups were described with means and standards. And independent *t*-tests were used to compare the demographic characteristics and scores of the pre-test of two groups to investigate whether there was a difference between the two groups before the intervention. Meanwhile, differences between the two groups in knowledge after experiment, learning satisfaction, and self-evaluation were analyzed by independent t-test and ANCOVA analysis as well. All statistical analyses were performed with the R (version 4.31) software, the Microsoft Office 2019, and IBM SPSS for Windows 27. And *p*-values less than 0.05 were considered statistically significant.

## Results

### Participant characteristics

Table 2 offers the baseline information of the participants. All the participants of the study were chosen from the same medical university, and their age ranged from 21 to 23, and *t-*test indicated that there was no significant difference between two groups (*p* = 0.32). All participants completed the experiment, and there was no dropout during the experiment. The average grades of the experimental and control group were 83.08 ± 4.87, and 82.00 ± 6.27, and the independent samples *t*-test indicated that there was no significant difference between two groups (*p* = 0.16). In addition, there was also no statistically significant difference in gender ratio and the grades of pre-test of two groups (all *p* > 0.05), using one way ANOVA analysis.

**Table 2:** Baseline characteristics of participants between the two groups

Characteristics	Categories	Blended learning (*n* = 37)	Traditional teaching (*n* = 39)	Statistics (*t*/*χ*^*2*^)	*p*
Age: Mean (SD)		22.49 (1.33)	22.21 (1.13)	0.98	0.322
Gender: n(%)	Female	12 (32.43)	15 (38.46)	0.55	0.583
	Male	25 (67.57)	24 (61.54)		
^a^Average grades: Mean (SD)		83.08 (4.87)	82.00 (6.27)	0.84	0.164
Pre-test grades: Mean (SD)		84.03 (4.83)	84.08 (4.32)	0.05	0.962

^a^Average grades: the average grades of the two groups in the semester before the study was carried out

### Comparison of learning outcome variables between the two groups

As is mentioned above, no significant difference was found between the two groups in pre-intervention variables. 76 questionnaires were distributed and 76 were effectively received with an effective recovery rate of 100%. We compared the variables of learning outcome of the two groups, including the grades of post-test, learning satisfaction, and self-evaluation (Table 3). The post-test score of the blended learning and traditional teaching groups were 88.08 ± 3.28 and 86.08 ± 2.74, respectively. We performed ANCOVA analysis in the post-test score and changes of score of the two groups, using pre-test score as covariate, the result showed that statistical significance between two groups on the post-test score and changes of score after adjusted by pre-test scores (*p* < 0.001). And statistical differences were found by *t*-test for the difference between the twice test scores of the two groups (*t* = 2.49, *p* = 0.022), which indicated that compared with traditional teaching, blended learning can significantly improve the learning outcomes of the students.

**Table 3:** Comparison of learning outcome variables between the two groups

Variables	Blended learning Mean (SD)	Traditional learning Mean (SD)	Statistics (*F*/*t*)	*p*
Post-test	88.08 (3.28)	86.08 (2.74)	38.21	0.001
Changes of score	4.05 (4.26)	2.00 (2.85)	82.06	0.001
Learning satisfaction	66.84 (2.04)	59.49 (3.01)	12.41	0.001
Self-evaluation	23.57 (0.93)	21.74 (1.09)	7.82	0.001

**Fig. 2:**
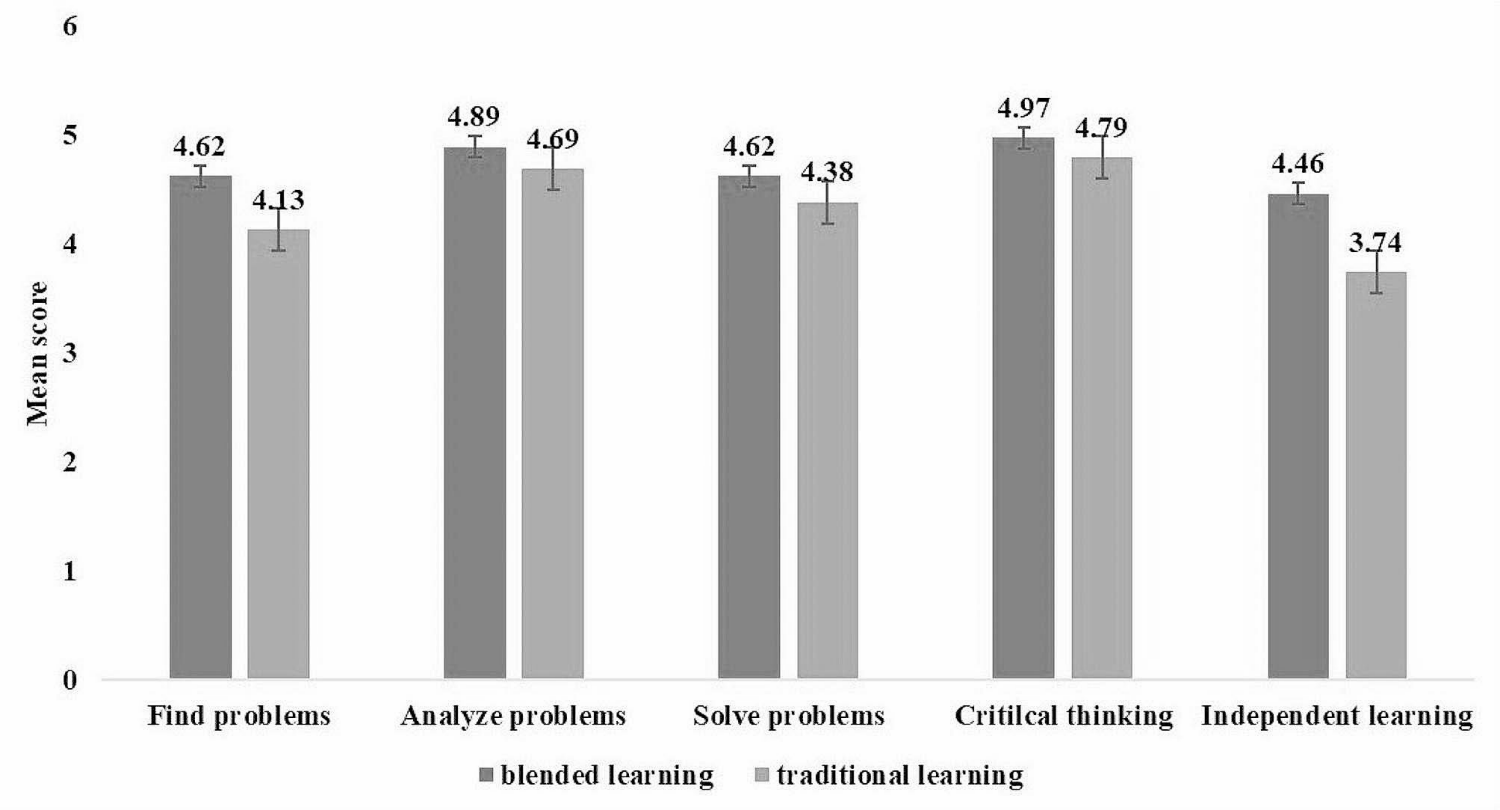
Mean scores of student’s self-evaluation

Furthermore, learning satisfaction of the blended learning group was significantly higher than the traditional learning group (*t* = 12.41, *p* < 0.001). Additionally, the self-evaluation score of the two groups also shows that the blended learning methods can produce higher self-evaluation scores (*t* = 7.82, *p* < 0.001), which indicated that blended learning methods can significantly improve the ability of problem-solving of the participants. Figure 2 showed that the blended learning group achieved higher score in every question than the traditional group in self-evaluation (*p* < 0.001). The result of *t*-test for all 14 questions of learning satisfaction showed that except for question 3, all the questions achieved higher scores in blended learning group (Table 4). Further analysis revealed that statistically significant difference was found on all 5 questions of self-evaluation(*p* < 0.05).

**Table 4:** Comparison of learning satisfaction between the two groups

Questions	Groups		*t*	*p*
	Blended learning Mean (SD)	Traditional learning Mean (SD)		
1	4.70 (0.52)	4.33 (0.66)	2.70	0.009
2	4.73 (0.45)	4.64 (0.54)	0.78	0.008
3	4.68 (0.48)	4.44 (0.60)	1.93	0.058
4	4.81 (0.40)	4.10 (0.75)	5.08	0.001
5	4.86 (0.35)	4.54 (0.51)	3.27	0.002
6	4.78 (0.48)	4.18 (0.76)	4.14	0.001
7	4.86 (0.35)	4.28 (0.79)	4.11	0.001
8	4.78 (0.48)	4.31 (0.77)	3.23	0.002
9	4.81 (0.46)	4.26 (0.75)	3.85	0.001
10	4.76 (0.50)	4.10 (0.85)	4.06	0.001
11	4.78 (0.48)	3.90 (0.88)	5.40	0.001
12	4.68 (0.48)	4.15 (0.78)	3.50	0.001
13	4.73 (0.51)	4.13 (0.73)	4.14	0.001
14	4.86 (0.35)	4.13 (0.73)	5.56	0.001

## Discussion

This study explored the influence of blended learning on learning outcomes in evidence-based medicine course. Based on the results of the study, after blended learning method was implemented, the learning outcomes of participants were significantly enhanced, including theoretical knowledge, learning satisfaction, and self-evaluation, the results were consistent with previous research on another course [12]. Especially, the changes of score between pre-test and post-test of two groups, the scores of participants from blended learning groups improved by 4.05 (4.26), while traditional learning group improved by 2.00 (2.85), indicated that the blended learning method can significantly improve students’ theoretical knowledge acquisition than traditional learning, which is consistent with several previous studies that focused on medical courses [23–25]. Participants of blended learning group learned independently in advance, which is the essence of the methods: learning first and then teach, and also make lectures can be not just knowledge imparters, but also a guide and edifier [22]. Moreover, blended learning group has been provided more multimedia resources to review and test the knowledge than the traditional learning group, which also contributed to the difference between the two groups.

On the aspects of learning satisfaction, it is noted that the scores of most questions were found statistically significant difference between two groups, except Q2 and Q3, with *p* values of 0.439 and 0.058 from *t* test, respectively. Question 2 was “Do you consider the class content is helpful for your major learning?”, and the participants of the study were from multiple disciplines pertain to medical specialty, which may result in no statistically significant difference. Question 3 was “How do you think the content of the class is interesting?”, the non-statistical significance of the results may be due to the selection of the basic content of evidence-based medicine in the teaching content. Natheless, in the total score of learning satisfaction blended learning group is significantly higher than traditional learning group, indicated that the blended learning methods can attain higher learning satisfaction, which is consistent with previous studies [26, 27]. Furthermore, blended learning group obtained significantly higher satisfaction on teaching design, classroom interaction, teaching environment set, which indicated that blended learning was superior in student-centered teaching, and can make students become the main body of classroom implementation and improve student’s classroom participation and learning effect.

Moreover, regarding self-evaluation, it is worth noting that although the results of all 5 questions were found statically different between two groups, *p* value from *t* test of Q2 was 0.048, which is very close to 0.05. Question 2 was “Do you think your ability to analyze scientific problems has improved through the study of this course”, and the chapters of the study were chosen from evidence-based medicine, only including find question and evidence, this may be the reason of *p* value of Q2 close to 0.05. A study from South Korea based on public healthcare education course indicated that blended learning method was effective in enhancing participants’ problem-solving abilities (*p* < 0.001), using a scale comprises 45 items developed by the Korean Educational Development Institute [12]. Our study only used 5 questions to assess the problem-solving ability of the participants, which may have caused the deviation.

Considering that the participants from the two groups were from two different grades, which may causer potential bias, as a result, reduce the credibility of the results, we compared the average grades and baseline information of the two groups in the semester before the study was carried out, and no statistically significant difference of the grades of pre-test was found, which effectively ensured the equilibrium and comparability of the two groups. Furthermore, the implementation of course teaching of the two groups was both in the first year of graduate students, which effectively avoid bias may be caused by the courses that have been studied, the learning and scientific research ability and cognitive level of participants. Moreover, the design of this study contained “internet plus” and flipped classroom, which is the mainstream model of the implementation of blended learning [12, 28–30]. Furthermore, we ordered different questions based on the same knowledge in the pre-test and post-test, which effectively avoided memory and selection bias.

Our study still had several limitations. Firstly, compared to traditional learning, the blended learning required teachers input on transition of the learning pattern, and its effectiveness was affected by the time, energy, and especially experience invested by the teachers which might cause certain bias in the results. To address this issue, the teachers’ team were kept the same for the two groups to reduce the potential influence. Furthermore, to carry out blended learning, pre-class preparation and classroom implementation place great demands on the quality and ability of the teachers, hence it is vital to cultivate teachers who can constantly adjust the teaching plan according to the feedback of students [22]. Moreover, “internet plus” raises requirements for classroom equipment, including projectors, screen, appropriate accessories including complex lighting and other technological issues, which has caused some difficulties for the implementation of blended learning [31, 32].

The first step of implementing blended learning is to be fully prepared before class, hence, it is important to provide appropriate resources such as videos and handouts for students. Future studies should focus on providing richer learning resources for students, such as massive open online course (MOOC) and Micro course. Furthermore, the team of teachers should explore more diversified teaching methods, including concept map, micro-class, and case-based teaching method [33–35], to improving learning outcomes.

## Conclusion

The results of the study showed that blended learning can effectively improve participants’ learning outcome, including test-score, learning satisfaction, and self-evaluation. Especially, in the terms of scores improvement, the results indicated that blended learning methods can enhance the performance of students significantly compared with traditional learning. Using blended learning method including “internet plus” and flipped classroom is recommended in the teaching of evidence-based medicine course.

## Data Availability

The datasets generated during and analyzed during the current study are available from the corresponding author.
